# A cross sectional comparison of postnatal care quality in facilities participating in a maternal health voucher program versus non-voucher facilities in Kenya

**DOI:** 10.1186/s12884-015-0588-y

**Published:** 2015-07-24

**Authors:** Charlotte E Warren, Timothy Abuya, Lucy Kanya, Francis Obare, Rebecca Njuki, Marleen Temmerman, Ben Bellows

**Affiliations:** Population Council, 4301 Connecticut Avenue NW, Washington DC, 20008 USA; Population Council General Accident Insurance House, Ralph Bunche Road, PO Box 17643, Nairobi, 00500 Kenya; Department of Obstetrics and Gynecology, Faculty of Medicine and Health Sciences, Ghent University, De Pinte Laan 185, B-9000 Ghent, Belgium

**Keywords:** Postnatal care, Vvoucher, OBA, Quality of care, Structure, Process and outcome

## Abstract

**Background:**

Health service fees constitute substantial barriers for women seeking childbirth and postnatal care. In an effort to reduce health inequities, the government of Kenya in 2006 introduced the output-based approach (OBA), or voucher programme, to increase poor women’s access to quality Safe Motherhood services including postnatal care. To help improve service quality, OBA programmes purchase services on behalf of the poor and marginalised, with provider reimbursements for verified services. Kenya’s programme accredited health facilities in three districts as well as in two informal Nairobi settlements.

**Methods:**

Postnatal care quality in voucher health facilities (*n* = 21) accredited in 2006 and in similar non-voucher health facilities (*n* = 20) are compared with cross sectional data collected in 2010. Summary scores for quality were calculated as additive sums of specific aspects of each attribute (structure, process, outcome). Measures of effect were assessed in a linear regression model accounting for clustering at facility level. Data were analysed using Stata 11.0.

**Results:**

The overall quality of postnatal care is poor in voucher and non-voucher facilities, but many facilities demonstrated ‘readiness’ for postnatal care (structural attributes: infrastructure, equipment, supplies, staffing, training) indicated by high scores (83/111), with public voucher facilities scoring higher than public non-voucher facilities. The two groups of facilities evinced no significant differences in postnatal care mean process scores: 14.2/55 in voucher facilities versus 16.4/55 in non-voucher facilities; coefficient: -1.70 (-4.9, 1.5), *p* = 0.294. Significantly more newborns were seen within 48 hours (83.5 % versus 72.1 %: *p* = 0.001) and received Bacillus Calmette-Guerin (BCG) (82.5 % versus 76.5 %: *p* < 0.001) at voucher facilities than at non-voucher facilities.

**Conclusions:**

Four years after facility accreditation in Kenya, scores for postnatal care quality are low in all facilities, even those with Safe Motherhood vouchers. We recommend the Kenya OBA programme review its Safe Motherhood reimbursement package and draw lessons from supply side results-based financing initiatives, to improve postnatal care quality.

## Background

An estimated 60 % of the world’s 270,000 annual maternal deaths occur within 48 hours of delivery, and two thirds of the three million annual newborn deaths occur within the first week [1, 2]. Early treatment in the postnatal period could save more lives and support adoption of healthy behaviours [3]. The postnatal period is largely neglected, however, and women and infants rarely receive optimal postnatal care [4].

Poverty and inequity are two primary underlying causes of maternal and newborn deaths in low and middle income countries [5]. Formal and informal fees for provision of healthcare during pregnancy, childbirth, and the immediate postnatal period constitute a substantial financial barrier for poor women [6–8]. To address these challenges, governments and donors have been prompted to find innovative ways of increasing access to maternal and newborn health services such as subsidised consumer led demand for quality services [9–12]. Vouchers and strategies incorporating supply and demand components, like the output-based approach (OBA), have demonstrated increases in service utilisation among targeted populations [6, 9, 11, 13–16].

A primary OBA or voucher programme objective is the increase of service utilisation by improving high priority populations’ purchasing powers, with a secondary objective of improving quality through reimbursements to providers for verified service delivery. Service reimbursements link pre-defined quality or quantity indicators to financial reimbursements, and in concert with competition with other providers or health facilities (public or private), motivate improvements in equitable access and quality of maternal and newborn health services including postnatal care [13, 17]. Other results-based or performance-based financing strategies focus on incentives for quality improvements that may not accelerate uptake of services among the poor and marginalised [18, 19].

Kenya’s maternal mortality ratio and newborn mortality rate are 488/100,000 live births and 31/1000 live births, respectively [20]. Over 90 % of women attend antenatal care at least once, 44 % give birth with a skilled attendant, and 47 % receive postnatal care [20]. Of the women who receive postnatal care, 42 % said they received a health check during the first two days, but no data provide information on the content of their postnatal care. Higher order births, and those in rural areas, are less likely to receive postnatal care than those of lower order and in urban areas. Generally, the most important characteristics of women associated with postnatal care are higher wealth statuses and having received antenatal care [20, 21]. Results from the 2010 Kenya service provision assessment indicate that only 59 % of facilities (from 34 % in the 2004 assessment) offer postnatal care services (compared to 74 % offering antenatal care). Although targeted postnatal care has reputedly increased emphasis on the first 48 hours after birth and includes check-ups through the first year, no specific module for postnatal care exists in the service provision assessment besides information in the antenatal care module [22].

### Financing strategy to improve quality of maternal and newborn health services in Kenya

The OBA project is intended to improve both the quality and access of appropriate maternal and newborn health services for the poor, through subsidized service purchase contracts with public and private providers [23–25]. Sold at highly subsidized prices, these vouchers provide direct subsidies to the poorest populations, permitting their access to Safe Motherhood services, and ultimately increasing their uptake, with qualified and certified service providers providing the services. The Ministry of Health (MoH) oversees implementation, supported by both the German Development Bank (KfW) and the Government of Kenya, with daily management, of voucher distribution, health facility claims and reimbursement, by a contracted private party as the voucher management agency [11, 13]. Criteria for accrediting public, private, and faith-based facilities were developed by the OBA project, adapted from existing national standards and guidelines, that are reviewed annually [23, 24].

Accredited health facilities are reimbursed for services for each voucher holder. The OBA programme provides participating facilities only with training for voucher reimbursement with the voucher management agency. In theory, health managers then use their disbursed funds to improve their facilities for a competitive advantage in encouraging use of their services (by both voucher holders and the general population) by procuring additional equipment, medicines, and supplies, along with building extensions and improving existing buildings, water sources, electricity, and other services, and updating their health care providers’ essential obstetric and newborn skills [26].

The voucher’s primary beneficiaries are economically disadvantaged women of reproductive age from Kisumu, Kitui, and Kiambu counties and two informal settlements in Nairobi County (Korogocho and Viwandani). Community distributors appointed by the voucher management agency sell the Safe Motherhood vouchers at a subsidised cost of 200 Kenyan shillings (equivalent to US$2.50). To identify poor women who qualify, distributors use a poverty grading tool assessing eight dimensions of household assets and amenities, expenditures and income, and health service access that are unique for each county. Any woman scoring eight to 16 points qualifies for the voucher. The design of the overall Kenya OBA voucher program is described in detail elsewhere [23, 24].

The Safe Motherhood voucher subsidises four antenatal visits, labour and delivery, and one postnatal visit. Safe Motherhood voucher clients are entitled to comprehensive essential obstetric and neonatal care including comprehensive newborn and post-delivery care [13, 23, 27]. The voucher programme offers two other vouchers: one for long acting and permanent family planning methods and the other for gender-based violence recovery services, but these are not discussed in this paper [11, 28].

Although evidence suggests that voucher programmes (including the Kenya programme) have potential for improving both reproductive health service utilisation [6, 10, 14] and quality of care [15, 16, 29, 30], none have specifically examined whether Safe Motherhood vouchers affect the quality of postnatal care, the most neglected element in the continuum of care. This paper attempts to fill that gap by comparing Kenya OBA programme health facilities, for four years, between 2006 and 2010, with a comparable cross-section of facilities without contracts for voucher clients (“non-voucher facilities”). This study hypothesizes that the quality of postnatal care in voucher facilities will be equal to or greater than the quality of those same services in non-voucher facilities [31].

## Methods

This paper uses data from a larger quasi-experimental design that evaluated the impact of the Kenyan OBA voucher scheme on selected reproductive health services’ quality and access. The larger study compared voucher-accredited health facilities with non-voucher facilities in counties with similar characteristics at two points in time (in 2010 and 2012); its study methodology is described in detail elsewhere [31]. For our analysis we used cross sectional data from 2010 to compare quality of postnatal care in health facilities accredited by the voucher programme since its inception in 2006 with similar non-voucher health facilities (a “post-test design”). We compare key outcomes from quality of postnatal care with similar non-voucher facilities.

This design was chosen due to the lack of random assignment of health facilities within intervention (voucher) or comparison (non-voucher) sites [31] because voucher sites were identified by MoH and KfW based on service gaps and need for increasing service coverage and availability for low income and remote populations. Facilities in targeted counties were asked to participate and were contracted if they met the accreditation criteria. Population Council, in conjunction with MoH, identified neighbouring counties with similar non-voucher facilities to maximise the likelihood of similar social, cultural, and economic characteristics in addition to similar reproductive health and healthcare behaviours among women 15 to 45 years old to serve as the comparison group [31]. Comparison facilities’ characteristics were similar to voucher facilities in their type of practice, available professional skills, clientele, locations, fees, and services including their levels (hospital, nursing home or health centre) and ownership type (public, private, faith based or non-governmental) [31].

### Study procedures

This paper uses quantitative data from health facility assessments in 41 health facilities (20 public and 21 private-for-profit or faith-based). The 21 voucher facilities were randomly selected from 56 accredited health facilities in the three counties (Kiambu, Kisumu, Kitui) and compared with 20 facilities from three non-voucher counties (Nyandarua, Uasin Gichu, Makueni). The health facility assessments used four study instruments:

#### Facility inventories

All inventories included a checklist for availability of infrastructure, equipment, commodities, medicines and supplies, as well as number of staff and services provided, along with provider training for staff (*n* = 41).

#### Interviews with 163 maternal health care providers

All maternal and newborn health providers working in facilities during data collection were approached for permission to interview them. Based on normal staffing levels, we expected four to eight providers eligible for interviewing in hospitals, and between two and four at health centres, for approximately 80 providers in each group and a total of 160. A total of 90 providers from voucher facilities and 73 in non-voucher facilities were interviewed. Providers were asked questions on their technical competence and time utilisation during the postnatal period.

#### Observations of 794 client-provider interactions

Client-provider interaction includes both a consultation’s process (how clients are treated and whether they actively participate) and content (what they are told, along with technical competence, accuracy of information and provision of essential information). Trained research assistants with clinical backgrounds observed postnatal consultations, assessing the consultation process with a standardized checklist. Subsequent sessions were observed, after patients’ informed consent, for 18 postnatal care clients in each facility (a total of 360 expected). A total of 499 postnatal care consultations in voucher facilities and 295 in non-voucher facilities were observed. Fewer clients in non-voucher facilities attended during the data collection period.

#### Exit interviews with 728 postnatal clients

Research assistants interviewed women as they exited their postnatal care consultations, focusing on the services, information, and counselling they received, as well as their fertility desires and postpartum family planning. A total of 484 exit interviews were conducted in voucher facilities, and 244 in non-voucher facilities.

### Ethics

Ethical approval for the evaluation was granted by Population Council’s Institutional Review Board (IRB) No. 470 and Kenya Medical Research Institute (KEMRI) SCC 174. All women attending PNC services during the data collection period were asked written permission to observe their consultation and to be interviewed afterwards. Written informed consent was obtained prior to all interviews that were conducted in settings that ensured privacy and confidentiality. Participants were informed they could withdraw from the research at any time. Data collectors were trained on ethical conduct.

### Quality of Care framework and development of scores

A study definition and framework for quality of care was adapted from Donabedian and Bruce using three general elements of quality: structure, process, and outcome [32, 33]. The elements of quality of care were assessed using health facility inventories, provider interviews, client–provider interactions, and client exit interviews (Table 1). Total scores were developed for the essential components of quality care. A list of minimum equipment required for postnatal care services, for example, was developed based on MoH guidelines [34, 35] and consultations with service providers: a working blood pressure machine; stethoscope; spotlight, flashlight or examination light; examination couch; baby weight scale; adult weight scale; and autoclave or other sterilizer.

**Table 1:** Quality of Care Framework

Structure	Process	Outcome
Data source: Facility inventory and providerknowledge	Data source: Observations of client – provider interactions	Data Source: Client exit interviews and service statistics
Facility inputs	QoC	Reduction in
Appropriate availability of services	• Quality of clinical care	• Waiting time
• Facility readiness	• Interpersonal care/rapport	Improvement in:
• 24 h availability	• History taking	• Interpersonal relations
• Emergency preparedness	• Range of services offered	• Time spent with provider
• Equipment, medicine and supplies	• Maternal health care	Client understanding
•Infection prevention	• Infant health care	• Client choice:
•Infrastructure	• Danger signs for mother and infant	• RH goals and family planning
• IEC materials available	• Family planning	• Knowledge
• Guidelines and registers	• HIV services	• Increase in:
Technical Competence	Information given to client	• FP uptake
•Education and Training	• Assess client understanding	• Infant immunisation uptake
• Supervision	• Documentation	• Satisfaction
• Provider knowledge		• Confidentiality/privacy
		• Continuity of care/followup
		Increase service provision
		• Range of services
		• Multiple service use
		• Diversification of client profile

To develop an overall quality score, a composite scoring system was generated by combining several indicators into a single score [36]. There are two methods for generating composite quality scores. The “Opportunity Model” is based on the percentage of functions (“quality indicators”) actually performed compared to the total number of targeted functions [37]. If a total of 125 functions, of a targeted total of 250, were performed on 10 clients, then the composite Opportunity Model quality score would equal 0.5 (=125/ 250). Typically, equal weighting is assumed for each function, which helps derive an aggregate composite quality score covering all functions. The second scoring method employs a pre-defined criteria system, the “Grading Model”. For example, in Zambia a national assessment of the quality of antenatal care classified all health facilities into three grades, “optimum”, “adequate”, or “inadequate”, using a set of criteria for access to care, responsiveness and appropriateness, continuity of care, patient safety, and effectiveness and efficiency [38]. We have used the Opportunity Model due to its ease of interpretation, with detailed scores for each function, group of functions, and aggregate. The Opportunity Model allows for easier comparison both within categories (e.g., indicators or groups of indicators) and among groups (e.g., facilities, sub-counties), whereas the Grading Model would make differentiation of two groups with the same grade difficult.

To measure the readiness of a facility to provide quality postnatal care, its attributes (infrastructure, equipment, commodities, drugs and supplies required for anyone to provide comprehensive postnatal care) are added, with equal weights, to create a structural score with a maximum of 107 points. The facility inventory study tool recorded these scores as well as each facility’s human resource elements including staffing numbers and availability of appropriate services. An additional scoring section, with a total of 32, assessed provider knowledge and training on maternal and newborn healthcare and postpartum family planning (including technical updates), with data drawn from provider interviews. The provider score was incorporated into the facility’s total score, for a potential composite score of 139 per facility.

Process attributes for quality, based on national and international standards [34, 39, 40] were analysed using data from our observations of client–provider interactions during postnatal consultations. Using the methodology described facility scores, summary process attributes for technical competence with equal weights (total score of 47) were derived from the observation checklists of client-provider interactions. These observations included how a provider performed in history taking, physical examinations of mother and baby, and counselling on maternal and newborn danger signs, return to fertility and healthy timing and spacing of pregnancies, infant feeding, and HIV and STI risk assessment and management, as well as consultation documentation. Interpersonal relations (with a score between 0 and 8) including privacy, confidentiality, and rapport between client and provider were also captured (Table 2).

**Table 2:** Attributes of care: structure, process and outcome

Attributes of quality	Elements assessed
Structure attributes: Infrastructure equipment and supplies (0–107)	
FP commodities available (score from 0–11)	Combined pill, Progestin only pill, Emergency Contraceptives, Injectables, IUCD, implants, male and female condoms, male and female sterilization, fertility based methods.
Testing reagents available (0–12)	Reagents for HIV (Elisa HIV-1 and Elisa HIV-2) Rapid reagents for HIV testing, Reagents for anaemia test, TB, urinary tract infections, malaria, cervical cancer screening and pregnancy test
General supplies (0–6)	Disposable needles and syringes, Insecticide treated nets, specimen bottles/pots for urine, sputum and blood, slides for malaria parasites
Essential vaccines (0–6)	Tetanus Toxoid, BCG, Measles, Polio, Hepatitis B, Pentavalent
ARV Drugs (0–7)	Nevirapine tabs and syrup, Zidovudine (ZDV, AZT),AZT syrup, Stavudine, Zidovudine + Lamivudine (Combivir),Miconazole or clotrimazole pessaries,
STI and RH drugs (0–10)	Ciprofloxacin oral, Erythromycin oral, Tetracycline oral, Benzathine Penicillin, Cotrimoxazole tabs and syrup, Metronidazole tablets, Metronidazole IV, Gentamicin IV, Amoxycillin
Infection control supplies (0–9)	Sterile and clean latex gloves, clean non-latex gloves, decontamination solution, waste receptacle with and without lid and plastic liner, container for used sharps, single use hand drying towels or a functioning electric hand dryer, running water
FP equipment/supplies (0–18)	speculum (small/medium/ large), tenacula, troca, surgical scissors, kidney dishes, sponge holding forceps, mosquito forceps – curvedand straight, surgical blade: size 15/11, draping towels, betadine, gauze, elastoplast
Delivery supplies/kits (0–5)	Delivery kit, Suture kit, Minilap BTL kit, Foetal scope, MVA kit
General equipment (0–7)	A working blood pressure machine, stethoscope, spotlight or flashlight orexamination light, examination couch, functional weighing scales for babiesand adults, autoclave/ sterilizer
Emergency equipment (0–5)	Oxygen, Adult and newborn resuscitation set Magnesium Sulphate, Calcium gluconate.
General infrastructure/utilities (0–11)	Waiting area is shaded and with seats, Waiting area for new admissions, Heater for delivery room and nursery, Functioning delivery bed, Postnatal ward, Private space for FP, ANC and PNC examination, 24 h supply of clean water and power to ensure fridge remains functional, Reliable lighting, Client toilets, Clean water for drinking with clean cups/glasses.
Structure attributes: Provider knowledge and training (0–32)	
Updates in last 24 months (0–7)	Family planning, contraceptive technology updates, targeted postnatal care, PMTCT, screening for cervical cancer, EONC.
Knowledge of postnatal care (0–5)	Routine health care for mother and baby, return to fertility, family planning counselling, infant feeding, immunizations
Knowledge of maternal danger signs (0–7)	Foul smelling vaginal discharge, heavy vaginal bleeding, severe lower abdominal pain, fever with or without chills, swollen hands, face, legs, severe headache and/or blurred vision. excessive tiredness or breathlessness,
Knowledge of basic preventive newborn care(0–7)	Early initiation of and exclusive breastfeeding for 6 months, early detection of problems/anger signs, clean delivery practices, warmth, cord, eye care.
Knowledge of danger signs in newborn (0–6)	Poor or no breastfeeding /feeding, difficulty breathing, hypothermia or hyperthermia, septic spots/boils on body, restlessness or irritability, jaundice.
Process attributes: Provider technical skills (0–47)	
History taking (0–7)	Date of delivery, if resumed menses, about HIV status, about medication currently taken, place of delivery, mode of delivery, if currently breast feeding.
Physical examination (0–7)	Took client’s temperature and blood pressure, check for pallor (anaemia), examine breasts and nipples, palpate the client’s abdomen for uterine involution, checked perineum and discharge/lochia, checked extent of PV bleeding.
Danger signs advice given (0–3)	Excessive vaginal bleeding, fever with or without chills, broken scars(Perineum/Caesarean).
Fertility advice given (0–5)	Discuss return to fertility, discuss healthy timing and spacing of pregnancies, discuss the health benefits for mother and baby when birth spacing, resume sexual activity and discussion of any method.
STI/HIV risk assessment (0–3)	STI with the client, HIV/AIDS with the client, STI and/or HIV risk factors with the client.
STI/HIV risk factors (0–4)	Multiple partners, STIs increase risk of HIV, unprotected sexual intercourse, not knowing partner’s status.
STI management (0–3)	Give information on symptoms of an STI, screen for STI, advise to seek medical treatment if they notice STI symptoms.
Infant feeding advice (0–3)	Discussed infant feeding, discuss how mother was managing with breastfeeding, re-emphasize exclusive feeding (either breast or replacement).
Infant examination (0–4)	Examine baby (undressed), check temperature, check baby’s respirations, baby weighing.
Infant danger signs discussed (0–4)	Feeding difficulties - not sucking or sucking poorly, breathing difficulties, body feels hot or too cold, jaundice.
Documentation (0–4)	Provider looked at client’s health card before beginning the consultation/ while collecting information/examining the client, has a post-partum register, recorded information in register/tally sheet wrote on the client’s card.
Process attributes: Provider interpersonal skills(0–7)	
Rapport (0–7)	Greets client, used clients name, introduces herself, tells client what will be done, Encourages client to ask question, ensured privacy, assures confidentiality, courteous to client throughout.
Outcome attributes: Client experiences and services received	
Family planning uptake within 0–10 weeks	Proportion of clients receiving preferred methods.
Infant immunisation	Proportion of newborns received BCG
Waiting time (average)	Wait before seeing provider.
Time spent with provider	Length of consultation.
Baby or mother seen by provider	Within 48 h, between 3–7 days, between 1–2 weeks’ between 3–6 weeks, more than 6 weeks.
Satisfaction	Satisfied, somewhat satisfied or not satisfied at all with services received.

Outcome attributes focus on postnatal clients’ experiences and perceptions of their quality of care, including waiting times, perceptions of respect accorded, their understanding and knowledge of their health statuses, whether they asked questions during their consultations, opportunity to ask questions, range of services received for both mother and baby, and mechanisms encouraging follow up appointments (Table 2).

### Data analysis

Quantitative data were double entered using Epidata and exported to Stata 11 for analysis. For each component of quality of care—structure, process and outcome—summary scores were calculated as the sum of items representing specific aspects of each attribute (as defined above), and these demonstrate the overall quality score. Two-tailed unpaired t-tests with unequal variance evaluated group differences in the average process scores comparing intervention and comparison groups. A p-value of less than or equal to 0.05 was the threshold for significance. Pearson’s chi-square tests were used to evaluate differences in proportions for various patient-reported outcome measures that included waiting times to see providers, time spent with providers, and patients reporting satisfaction.

To assess measures of effect, a linear regression model was used for individual and summative process quality score outcomes. We controlled for clustering at facility level, type of provider defined as public or private, and level of care (hospitals and sub-district hospitals versus dispensaries, nursing homes and clinics). In all instances we report coefficients with their 95 % confidence intervals and *p* values for the coefficients for all three models.

## Results

### Characteristics of women attending postnatal care

The average ages (25.6 years) of postnatal women observed and interviewed were similar at voucher (*n* = 451) and non-voucher facilities (*n* = 237). There were no signifcant differences [*p* = 0.327] in education between the two groups, with most women reporting completion of primary (51.9 %) or secondary school (31.6 %). Around 82.2 % of women attending voucher facilities were married, compared to 87 % of women at non-voucher facilities [*p* = 0.078] (data not shown). There were no signficant differences between the poorest women (bottom two quintiles) attending voucher facilities and non-voucher facilities (32.6 % versus 39.3 %; *p* = 0.077). Significantly more women at voucher facilities said they would find it very difficult (31.3 % versus 9.5 %) or difficult (50.1 % versus 45.7 %) than women at non-voucher facilities to pay a health bill of more than 1,000 Kshs (US$12) [*p* < 0.001] (data not shown in tables).

Fifty seven percent of voucher facility clients used a Safe Motherhood voucher. The two primary reasons for women’s attendance for postnatal services were care for themselves and immunisations for their babies. Significantly more women at voucher facilities attended for infant immunisations than woman at non-voucher facilities (64.4 % versus 60.7 %; *p* < 0.001), with fewer attending for postnatal care services (19.5 % versus 25.6 %; *p* < 0.001) .

### Characteristics of health facilities

Facilities assessed are described in Table 3. The staffing cadres in the two facility groups were similar besides the fact that more medical doctors were available at voucher facilities. Postnatal consultations were generally performed by two cadres of nurses. There were no significant differences (*p* = 0.503) for registered nurses or midwives conducting postnatal care consultations at voucher facilities (68.0 %) and non-voucher facilities (61.3 %), but significantly fewer enrolled nurses or midwives conducted consultations at voucher facilities (23.1 % versus 30.6 % *p* = 0.021).

**Table 3:** Characteristics of study facilities

Key Features	Voucher facilities	Non-voucher facilities	Total	*P* value
Facility type	*n* = 21 (%)	*n* = 20 (%)	*n* = 41 (%)	
Hospital	15 (71.4)	12 (60.0)	27 (65.8)	0.162
Health centre	4 (19.0)	8 (40.0)	12 (29.2)	
Nursing home	2 (9.5)	0 (0.0)	2 (4.8)	
Sector				
Public	7 (33.3)	13 (65.0)	20 (48.7)	0.043
Private (NGO/Faith based)	13 (66.7)	7 (35.0)	21 (51.2)	
Number of providers available and working in MCH/FP, Maternity unit, ART				
Specialist doctors	36	45	81	Not significant
Medical officers	45	16	61	
Clinical officers	46	50	96	
Registered nurses/midwifes	204	146	350	
Enrolled nurses/midwives	118	103	221	
Laboratory technologist /technicians	11	24	35	
Pharmacists/technicians	4	16	20	
Nutritionists	20	16	36	
associated medical staff	9	2	11	
Lay counsellors	41	10	116	
Administrative staff	12	21	33	

### Structural attributes of quality: supplies and commodities

**Fig. 1:**
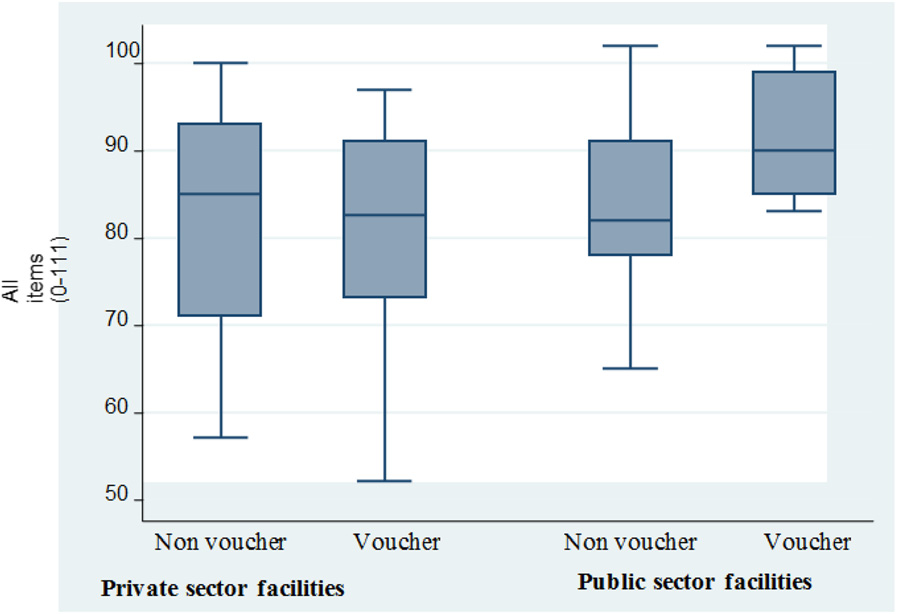
Readiness score for structural attributes available (0–107): infrastructure, equipment, medicines commodities and supplies. Legend: Box plot showing summary scores for structural aspects of care (equipment, supplies, staffing, training) for private and public sector health facilities

Overall there were no significant differences for availability of infrastructure, staffing, equipment, family planning commodities, medicines, and supplies in all facilities (Table 4). For the 111 features assessed, voucher facilities demonstrated 69.4 % compared to 68.8 % in non-voucher facilities. Additional analysis with a linear regression model shows non-significant scores, with voucher facilities scoring 2.4 points more than non-voucher facilities (coefficient 2.49: 95 % CI (-5.29, 10.28); *p* = 0.520). Public voucher facilities had higher mean scores than non-voucher public facilities, 91.7 versus 83.2; *p* = 0.091, with the regression model indicating a significant difference of 9.24 points higher than non-voucher facilities (coefficient 9.24: 95 % CI (1.3, 17.2); *p* = 0.024, illustrated in Fig. 1.

**Table 4:** Basic infrastructure and provider training: structural attributes

Mean score of facilities with the following equipment /supplies (SD)^a^:	Voucher facilities (*n* = 21)	Non-voucher facilities (*n* = 20)	Total (*n* = 41)	*P* value
	n SD	n SD	n SD	
FP commodities (0–11)	7.7 (2.6)	8.2 (2.5)	8.1 (2.6)	0.590
Testing reagents (0–12)	10.0 (1.9)	9.9 (2.1)	9.5 (2.1)	0.814
General supplies (0–6)	5.2 (0.8)	5.6 (0.6)	5.5 (0.8)	0.179
Essential vaccines (0–6)	5.2 (0.7)	4.8 (1.4)	5.0 (0.9)	0.122
ARV Drugs (0–7)	6.1 (1.3)	5.5 (2.5)	5.9 (1.8)	0.300
STI and RH drugs (0–10)	4.2 (3.7)	4.6 (3.5)	5.3 (3.6)	0.719
Infection control supplies (0–9)	7.2 (1.3)	7.1 (1.3)	6.7 (1.4)	0.655
FP supplies (0–18)	14.8 (3.9)	14.6 (3.5)	14.2 (3.6)	0.891
Delivery supplies and kits (0–5)	3.4 (1.2)	3.5 (1.1)	3.2 (1.1)	0.935
General equipment (0–6)	5.9 (0.3)	5.7 (0.6)	5.7 (0.5)	0.323
Emergency equipment and drugs (0–4)	3.0 (1.1)	3.3 (0.8)	3.0 (0.9)	0.329
General infrastructure (0–11)	10.1 (1.1)	9.8 (1.3)	9.5 (1.5)	0.305
Total 0–111 (SD)	83.3 (13.4)	82.6 (12.1)	82.9 (12.6)	0.855
Provider training and updates				
% of providers receiving training in the last 24 months on:	90	73	163	*P* value
	n %	n %	n %	
Family planning	26 (28.9)	19 (26.0)	45 (27.6)	0.684
Contraceptive technology update	27 (30.0)	22 (30.1)	49 30.1	0.985
Targeted PNC	21 (23.3)	9 (12.3)	30 (18.4)	0.078
PMTCT	37 (41.1)	30 (41.1)	67 (41.1)	0.998
Screening for cervical cancer	28 (31.1)	12 (16.4)	40 (24.5)	0.030
Newborn care	24 (26.7)	18 (24.7)	42 (25.8)	0.771
Essential obstetric care	22 (24.4)	21 (28.8)	43 (26.4)	0.562

^a^ *SD* Standard deviation

### Structural attributes of quality: provider knowledge and reports on information they provide to postnatal women

Providers said they received training on relevant maternal and newborn care, with no difference between facility groups (Table 4). Minimal overall differences were observed for providers’ knowledge of postnatal care aspects in voucher and non-voucher facilities. While assessing reported provider practice, significantly more providers at voucher sites (than non-voucher facilities) reported counselling women on the importance of early initiation of breastfeeding (54.0 % versus 38.4 %: *p* = 0.048) during postnatal consultations. There were few differences between providers’ reports of what maternal danger signs they advise postnatal women of, apart from significantly more voucher facility providers reported counselling on signs of severe pre-eclampsia (44.2 % versus 26.0: *p* = 0.017) in the immediate postnatal period (first 2 to 3 days after birth). Conversely, more providers at non-voucher facilities reported offering information on family planning to postnatal women (83.6 % versus 67 %: *p* = 0.017).

### Process attributes of care: technical aspects

Overall composite scores derived from our observations of consultations for postnatal care process attributes were generally low for both groups of facilities, with lower scores in voucher facilities, where 14.2 out of the 55 elements were demonstrated compared to 16.4 in non-voucher facilities, *p* = 0.0001 (Table 5). Regression analysis indicates that voucher facilities scored 1.7 % lower than non-voucher facilities, but this difference was not statistically significant; coefficient -1.70 (95 % CI) (-4.9, 1.5), *p* = 0.294 (Table 6). There were also no significant differences for public facilities versus private facilities, nor for hospitals versus lower level facilities such as health centres and dispensaries.

**Table 5:** Observed provider practices (process attributes)

Provider practices during postnatal care consultations (observed from client provider interactions)				
Mean scores for various constructs (SD)	Voucher consultations (*n* = 479)	Non-voucher consultations (*n* = 241)	Total (*n* = 720)	*p* values
	SD	SD	SD	
Maternal care				
History taking practices (0–7)	2.2 (1.7)	2.7 (1.8)	2.4 (1.7)	0.0008
Physical examination of the mother (0–7)	1.1 (1.8)	1.3 (1.9)	1.2 (1.8)	0.214
Advice on danger signs for the mother (0–3)	0.2 (0.5)	0.2 (0.5)	0.2 (0.5)	0.372
fertility advice (0–5)	1.4 (0.8)	1.7 (1.1)	1.5 (0.9)	0.001
STI/HIV risk assessment (0–3)	0.2 (0.6)	0.3 (0.6)	0.2 (0.6)	0.034
STI/HIV risk factors (0–4)	0.1 (0.6)	0.2 (0.5)	0.2 (0.6)	0.779
STI management (0–3)	0.02 (0.2)	0.02 (0.1)	0.1 (0.2)	0.812
Total for maternal care (0–32)	4.6 (4.3)	5.8 (4.4)	5.0 (4.4)	0.0004
Infant care				
Infant feeding advice (0–3)	1.4 (1.3)	1.9 (1.3)	1.5 (1.3)	<0.001
Infant examination (0–4)	1.0 (0.9)	0.9 (0.8)	0.9 (0.9)	0.275
Infant danger signs discussed (0–4)	0.2 (0.7)	0.4 (0.9)	0.3 (0.8)	0.004
Total for infant care (0–11)	2.6 (2.1)	3.2 (2.1)	2.8 (2.1)	0.002
Documentation				
Total for documentation (0–4)	2.9 (1.1)	2.9 (1.3)	2.9 (1.2)	0.796
Inter personal skills				
Total creation of rapport (0–8)	4.1 (1.4)	4.4 (2.0)	4.2 (1.6)	0.006
Total for process score (0–55)	14.2 (6.8)	16.4 (7.5)	14.9 (7.1)	0.0001

**Table 6:** Linear regression outputs for observed practices during postnatal care consultations

Constructs	Coefficients for various construct scores (95 % CI)					
Maternal care	Group (ref: voucher facilities)	*P* value	Sector (ref: public sector)	*P* value	Level of care: (ref: hospital)	*p* value
History taking practices (0–7)	-0.25 (-0.97, 0.45);	0.464	0.60 (-0.13,1.32)	0.103	-0.40 (-1.0,0.21)	0.191
Physical examination of the mother (0–7)	-0.10 (-0.82,0.61)	0.772	0.29 (-0.44,1.0)	0.427	0.16 (-0.49, 0.82)	0.618
Advice on danger signs for the mother (0–3)	-0.018 (-0.22, 0.18)	0.857	0.081 (-0.14,0.31)	0.479	0.078 (-0.09,0.25)	0.369
fertility advice (0–5)	-0.25 (-0.69,0.17)	0.239	0.54 (0.16.0.92)	0.006	0.11 (-0.24,0.47)	0.529
STI/HIV risk assessment (0–3)	-0.02 (-0.20,0.24)	0.844	0.27 (0.06,0.48)	0.011	0.12 (-0.04,0.29)	0.152
STI/HIV risk factors (0–4)	0.03 (-0.19,0.25)	0.783	0.16 (-0.09,0.42)	0.200	0.09 (-0.10,0.28)	0.346
STI management (0–3)	0.013 (-0.02,0.56)	0.520	0.036 (-0.009,0.083)	0.115	0.023 (-0.009,0.055)	0.156
Total maternal care (0–32)	-0.61 (-2.6.2.37)	0.535	2.00 (-0.019, 4.02)	0.052	0.18 (-1.45,1.83)	0.817
Infant care						
Infant feeding advice (0–3)	-0.40 (-0.97,0.15)	0.152	0.40 (-0.17,0.99)	0.167	0.31 (-0.31,0.94)	0.315
Infant examination (0–4)	0.027 (-0.27,0.32)	0.854	-0.16 (-0.51,0.17)	0.327	-0.003 (-0.31,0.30)	0.980
Infant danger signs discussed (0–4)	-0.12 (-0.38,0.13)	0.324	0.20 (-0.004,0.42)	0.055	0.10 (-0.12,0.53)	0.210
Total for infant care (0–11)	-0.51 (-1.29,0.27)	0.198	0.44 (-0.40,1.3)	0.296	0.42 (-0.52,1.33)	0.365
Documentation (0–4)	0.04 (-0.48,0.56)	0.877	0.048 (-0.41,0.51)	0.836	-0.01 (-0.58,0.55)	0.963
Interpersonal skills						
Total creation of rapport (0–8)	-0.62 (-1.43,0.18)	0.128	-0.96 (-1.61,0.32)	0.004	-0.50 (-1.22,0.21)	0.162
Total for process score (0–55)	-1.70 (-4.9,1.5)	0.294	1.53 (-1.4,4.5)	0.304	0.085 (-2.8,3.0)	0.954

For individual elements assessed in Table 5, non-voucher facilities appear to have higher scores for history taking, fertility advice, infant feeding advice, and infant danger signs discussed, although these scores were not statistically significant when controlled for clustering. Public facilities did have 0.54 point higher scores for STI and HIV risk assessment compared to private facilities; coefficient. 0.27 (95 % CI) (0.06, 0.48) *p* = 0.01 (Table 6).

### Process attributes: interpersonal skills

There were significant differences in average scores observed for rapport, with providers at voucher facilities scoring significantly lower than non-voucher facility providers (mean score 4.1 versus 4.4 out of 7: *p* = 0.006). There were individual variations on the seven aspects of building rapport, however. In voucher facilities more providers were observed using the client’s name, but fewer were observed greeting the client or ensuring privacy or confidentiality. While comparing performances of public and private providers, those in public facilities scored 0.96 lower than private providers for building rapport during postnatal care consultations; coefficient -0.96 (95 % CI) (-1.61, 0.32) *p* = 0.004 (Table 6).

### Outcome attributes

Table 7 demonstrates some significant differences for waiting times of women at voucher and non-voucher facilities, but not for time spent with their provider. Women attending postnatal care at voucher facilities were more likely to have delivered in a health facility than women at non-voucher facilities (88.7 % versus 77.8 %; *p* = 0.001) (data not shown in tables). More women (not significant) and newborns (significant *p* < 0.001) at voucher facilities were seen within 48 h of childbirth (Table 7). Only 16 % of women at voucher facilities and 17.7 % at non-voucher facilities were interviewed leaving the maternity unit, which indicates a high proportion of women returning to facilities to access care within 48 h of birth. Similar proportions of women received their preferred family planning methods, and most received injectables or progestin-only pills.

**Table 7:** Key outcome measures of quality of postnatal care (from exit interviews)

% of clients who	Voucher clients	Non-voucher clients	Total	*p* values
	*n* (%)	*n* (%)	*n* (%)	
Saw the provider within:	468	244	712	0.081
within half hour of arriving at facility	315 (67.3)	160 (65.6)	475 (66.7)	
30 min-1 h	37 (7.9)	13 (5.3)	50 (7.0)	
1–2 h	22 (4.7)	6 (2.5)	28 (3.9)	
above 2 h	94 (20.1)	65 (26.6)	159 (22.2)	
Took the following time with provider	468	244	712	0.674
1–5 min	67 (14.3)	28 (11.8)	95 (13.3)	
6–10 min	103 (22.1	61 (25.0)	164 (23.0)	
11–15 min	68 (14.5)	37 (15.1)	105 (14.7)	
16–30 min	68 (14.5)	40 (16.3)	108 (15.2)	
over half hour-3 h	162 (34.6)	78 (31.9)	240 (33.7)	
% of postnatal women who had undergone a first checkup:	259	146	335	0.314
Within 48 h	218 (84.2)	117 (80.1)	335 (82.7)	
Between 3 to 7 days	5 (1.9)	2 (1.3)	7 (1.7)	
Between 1 to 2 weeks	29 (11.2)	18 (12.3)	47 (11.6)	
Between 3 to 6 weeks	6 (2.3)	9 (6.2)	15 (3.7)	
more than 6 weeks	1 (0.4)	0 (0.0)	1 (0.3)	
Received preferred family planning method	23 (48.9)	17 (56.7)	40 (51.9)	0.508
% of infants who had undergone a first checkup:	426	229	655	
Within 48 h	356 (83.5)	165 (72.1)	521 (79.5)	0.001
Between 3 to 7 days	13 (3.1)	10 (4.4)	23 (3.5)	
Between 1 to 2 weeks	43 (10.4)	34 (14.8)	77 (11.8)	
Between 3 to 6 weeks	12 (2.8)	20 (8.7)	32 (4.8)	
More than 6 weeks	2 (0.4)	0 (0.0)	2 (0.3)	
% reporting that they	450	242	692	0.152
Satisfied with services	399 (88.7)	208 (85.9)	607 (87.7)	
Somewhat satisfied with services	34 9 (7.5)	28 (11.5)	62 (8.9)	
Not satisfied at all	17 (3.7)	6 (2.5)	23 (3.3)	

Significantly more women at voucher facilities reported immunisation of their newborns with BCG during the postnatal care visit than at non-voucher facilities (82.5 % versus 76.5 %: *p* < 0.001). Around 87 % of women said they were satisfied with their treatment on the day of interview. Vouchers were utilised for antenatal care (74.8 %) and delivery services (87.0 %), but fewer than half were used for postnatal care (48.5 %).

## Discussion

This analysis aims to understand the influence of Safe Motherhood vouchers on postnatal care quality by comparing public, private, and faith-based health facilities enrolled in the Kenya OBA program for four years (from 2006 to 2010) with a group of similar facilities with no access to vouchers. A quality of care framework adapted from Donabedian and Bruce grouped elements or attributes into structures, processes, and outcomes of postnatal care [32, 33]. Key indicators of interest include facilities’ readiness to provide postnatal care (structures), maternal and infant counselling and care during a postnatal consultation (processes), and services women or their newborns actually received, including their perceptions of the care they received (outcomes).

Guidelines for postnatal care in Kenya are described in national documents dating from 2004, with a specific postnatal care register disseminated in 2005, and in 2007 an orientation package for targeted postnatal care was developed and distributed [34, 39]. In 2010 59 % of Kenyan health facilities reported providing postnatal care [22] and accreditation criteria for facilities included provision of postnatal care. Our data show, however, that both voucher and non-voucher facilities scored below the expected scores for their overall postnatal care quality.

### Structure

In OBA programs it is often assumed that their minimum quality standards for accreditation and the expected competition between health facilities encourage providers to improve their quality of care. Voucher-accredited public facilities do appear to score higher for structural attributes than non-voucher public facilities and all private facilities. In another component of the study, the evaluation team interviewed facility managers to ascertain how they utilised their voucher remittances [41]. Most used their funds on structural improvements such as renovating maternity units, laboratory or operating theatres as well as purchasing drugs, equipment or supplies. Moreover, public health facilities were required to follow strict MoH procedures for disbursed funds with formal requests to their county managers prior to any expenditures. Public facilities were also not able to use the funds for recruiting additional staff or on skills training [41, 42]. This MoH policy may explain why public voucher facilities scored higher on structural attributes but lower on process [13, 41]. Slightly lower scores by private voucher facilities than private non-voucher facilities may provide positive evidence that vouchers were correctly targeted at low income women who visit less sophisticated facilities.

Providers’ knowledge and practice were also mixed. More voucher providers reported clients counselled on the importance of breastfeeding, but more non-voucher providers said they counselled women on family planning, which is unexpected due to the fact another voucher for family planning is available for poor women. From our review of facilities’ remittances we know that little is spent on provider training [41], and we recommend that providers (both public and private) have more access to training sessions and contraceptive technology updates organised by the Ministry of Health.

Although these high structural attribute scores could be taken as synonymous with availability or readiness to provide services and commodities, they are incomplete, because clinical or process attributes for the provision (and receipt) of comprehensive postnatal care are not included [32]. A 2010 review and design of an extension to the OBA programme also concluded that postnatal care had yet to change significantly by virtue of reimbursement funds alone [42].

### Process

All facilities’ composite scores totalled less than one third of the maximum score, well below the standards of postnatal care provision in the national guidelines [34, 43]. Although voucher facilities scored significantly worse than non-voucher facilities, no significant differences overall were observed for public and private facilities or hospitals and lower level facilities. Non-voucher facilities performed better in history taking and counselling on fertility and infant care, but these scores were not significant when controlled for clustering. Public facilities scored higher on counselling for HIV and STI risk assessment and family planning than private facilities, but private facilities had higher scores for rapport.

The overall low performance in clinical care (or process attributes) at both voucher and non-voucher sites could be explained by several factors. Although providers reported some clinical updates for a range of maternal and newborn care, it is possible providers were unable to translate this knowledge into practice [44]. Secondly, although women are entitled to postnatal care with the Safe Motherhood voucher, providers often only immunise an infant rather than providing a comprehensive package of care for both mother and child. The difficulty of conducting a check up for a mother in a busy immunisation clinic, without privacy or examination couches, can contribute to infant-focused care. Thirdly, women may not be aware of the comprehensive package of postnatal care, as over 60 % of women in this study came to postnatal care for infant immunisation. Elsewhere in the Kenya voucher evaluation we found communities with misunderstanding about what services vouchers actually cover [26]. Finally, although there are now separate vouchers for each of the four antenatal care visits as well as childbirth, there is no specific paper voucher for postnatal care, so facilities can only claim reimbursement for this service as an extension of the delivery voucher (i.e., if a woman give birth in a facility), but women are only encouraged to return to a facility after six weeks for infant immunisation. Nevertheless, it was expected that the OBA programme would improve the overall quality of maternal care including postnatal care.

If voucher facilities are not reimbursed for providing comprehensive postnatal care, this may hinder providers’ motivation for offering any postnatal services for mothers and infants, especially in private facilities that do not receive updates from MoH. There are clearly defined guidelines and standards about the content and timing of mother and infant care immediately after birth, at one or two weeks postpartum, and up to six weeks after birth. Many providers, specifically those from private facilities, appear to be unaware of these national postnatal guidelines, and this contributes to service discontinuity after pregnancy and delivery [3, 45].

### Outcomes

More than four fifths of all women were seen within 48 h, and significantly more newborns at voucher facilities received care in their first 48 h in addition to BCG vaccinations than those at non-voucher facilities. Evidence suggests that newborns seen within the first two days of birth by a provider have a greater chance of survival [46]. Overall, postnatal women seemed satisfied with their care, waiting times, and time spent with a provider, regardless of facility type. Clinical competence is less easily judged by clients, and often they evaluate providers on the amount of time spent with them, and their caring attitude, than on technical skills [32]. Although the output attributes based on clients’ experience may not necessarily translate into adequate clinical care, such information presents an opportunity for the health system to understand women’s perceptions of quality postnatal care and what motivates them to seek services.

A comprehensive postnatal care package should include routine visits in the days following childbirth, when risks are high for both mother and baby, complemented by promotion of healthy behaviours (e.g., exclusive breast feeding), for identifying complications and facilitating referrals [3]. Kenya’s MoH recommends one visit within 48 h of delivery, another within one week, a third between four and six weeks postpartum, and a fourth at six months [39]. Postnatal registers have been in existence in Kenya since 2005, but few facilities routinely record or collect this information, or report it through the government health information system. An attempt to deduce potential increased workloads associated with increased in attendance at voucher facilities and its effect on quality of care was not possible due to these recording gaps. The OBA programme’s voucher management agency is in the process of converting the paper vouchers to a ‘smart card’ that may capture all services offered to and received by voucher clients, including postnatal care.

A review of the literature on the quality of private and public health care in low and middle income countries indicates that improving quality of care in a health system is a long term effort and requires attention to various aspects including the incentive structure and providers’ training [47]. Supervision and clinical audits with resultant recommendations, especially if combined with training, have been found to be effective in improving quality [48]. The OBA project itself did not provide any technical training, and expected health facilities to improve their own staff skills through technical updates. Apparently this did not happen sufficiently, although providers received some related maternal and infant care training within the past 24 months.

For the adequate clinical care of postnatal women, and the diagnosis and early treatment of any complications, an evidence-based schedule for visits is critical. While Kenya has detailed the timing and content for postnatal visits in policy guidelines, it appears there is a policy and care provision gap: Many health care providers do not perceive the importance of the postnatal period, although four fifths of women interviewed sought care within 48 h postpartum. A community-based approach may be required to ensure that women discharged after delivery receive adequate information, support, and follow up. Providers should receive more training updates specifically for the postnatal period, and any training should be followed by supportive supervision. Policy guidelines should be more easily available for private and faith-based facilities.

In other results-based financing initiatives, supply side incentives reward improvements in structural and process indicators for quality. These programs develop verification mechanisms that routinely confirm service deliveries and measure quality indicators at contracted facilities. Most OBA voucher programs do not have an explicit financial incentive for quality improvements, and it might be worthwhile for the Kenya program to consider one [18].

There are limitations to this analysis. Its cross sectional design, coupled with a non-random facility selection, limits any tests for causality. Control facilities were, however, matched by administrative type and similar infrastructure, staffing and service characteristics, along with a qualitative assessment of each local healthcare market and access. Ideally, the voucher and non-voucher facilities would be alike in every way except for the OBA voucher contract. Because facilities differed according to ownership status it is possible other differing factors, such as financing and catchment size, could affect quality of care outcomes. Secondly, although comparison sites were carefully selected, support by other partners for maternal and newborn health may also have confounded results. We did control for clustering at facility level, provider type, and level of care. Thirdly, the study population of postnatal women attending a health facility is not representative of Kenya’s general postnatal population, where only 44 % of newly delivered women attend postnatal care [20]. These findings do, however, represent the services received by recently delivered women in most Kenyan health facilities.

## Conclusion

Overall, the quality of postnatal care in all facilities was low, which indicates that the postnatal period continues to receive limited attention from both women and providers, even where a Safe Motherhood voucher exists. The OBA voucher programme should include separate reimbursement for comprehensive postnatal care services to address this critical period. In addition, the voucher programme should explore incentives for quality improvement targets in each service subsidised by the programme.

## Abbreviations

ANC: Antenatal Care
BCG: Bacillus Calmette-Guerin
CPI: Client-Provider Interaction
HFA: Health Facility Assessment
IRB: Institutional Review Board
KEMRI: Kenya Medical Research Institute
KfW: German Development Bank
MNH: Maternal and Newborn Health
MoH: Ministry of Health
PNC: Postnatal Care
QoC: Quality of Care
OBA: Output-based Aid
SM: Safe Motherhood
STI: Sexually Transmitted Infection
VMA: Voucher Management Agency
